# Genome Assembly and Annotation of the *Trichoplusia ni* Tni-FNL Insect Cell Line Enabled by Long-Read Technologies

**DOI:** 10.3390/genes10020079

**Published:** 2019-01-23

**Authors:** Keyur Talsania, Monika Mehta, Castle Raley, Yuliya Kriga, Sujatha Gowda, Carissa Grose, Matthew Drew, Veronica Roberts, Kwong Tai Cheng, Sandra Burkett, Steffen Oeser, Robert Stephens, Daniel Soppet, Xiongfeng Chen, Parimal Kumar, Oksana German, Tatyana Smirnova, Christopher Hautman, Jyoti Shetty, Bao Tran, Yongmei Zhao, Dominic Esposito

**Affiliations:** 1 Advanced Biomedical Computational Science, Frederick National Laboratory for Cancer Research sponsored by the National Cancer Institute, Frederick, MD 21701, USA; keyur.talsania@nih.gov (K.T.); xiongfong.chen2@nih.gov (X.C.); 2 Cancer Research Technology Program, Frederick National Laboratory for Cancer Research Sponsored by the National Cancer Institute, Frederick, MD 21701, USA; monika.mehta@nih.gov (M.M.); castleraley@gwu.edu (C.R.); krigay@mail.nih.gov (Y.K.); sujatha.gowda2@nih.gov (S.G.); soppetdr@mail.nih.gov (D.S.); parimal.kumar@nih.gov (P.K.); oksana.german@nih.gov (O.G.); tatyana.smirnova@nih.gov (T.S.); christopher.hautman@nih.gov (C.H.); jyoti.shetty@nih.gov (J.S.); bao.tran@nih.gov (B.T.); 3 NCI RAS Initiative, Frederick National Laboratory for Cancer Research Sponsored by the National Cancer Institute, Frederick, MD 21701, USA; carissa.grose@nih.gov (C.G.); matt.drew@nih.gov (M.D.); veronica.roberts@nih.gov (V.R.); oscar.cheng@nih.gov (K.T.C.); stephensr@mail.nih.gov (R.S.); 4 Comparative Molecular Cytogenetics Core Facility, Frederick National Laboratory for Cancer Research sponsored by the National Cancer Institute, Frederick, MD 21701, USA; sandra.burkett@nih.gov; 5 Bionano Genomics, San Diego, CA 92121, USA; soeser@bionanogenomics.com

**Keywords:** de novo assembly, PacBio single molecule real-time sequencing, *Tricoplusia ni*, insect genome, next generation sequencing, optical mapping

## Abstract

Background: *Trichoplusia ni* derived cell lines are commonly used to enable recombinant protein expression via baculovirus infection to generate materials approved for clinical use and in clinical trials. In order to develop systems biology and genome engineering tools to improve protein expression in this host, we performed de novo genome assembly of the *Trichoplusia ni*-derived cell line Tni-FNL. Methods: By integration of PacBio single-molecule sequencing, Bionano optical mapping, and 10X Genomics linked-reads data, we have produced a draft genome assembly of Tni-FNL. Results: Our assembly contains 280 scaffolds, with a N50 scaffold size of 2.3 Mb and a total length of 359 Mb. Annotation of the Tni-FNL genome resulted in 14,101 predicted genes and 93.2% of the predicted proteome contained recognizable protein domains. Ortholog searches within the superorder *Holometabola* provided further evidence of high accuracy and completeness of the Tni-FNL genome assembly. Conclusions: This first draft Tni-FNL genome assembly was enabled by complementary long-read technologies and represents a high-quality, well-annotated genome that provides novel insight into the complexity of this insect cell line and can serve as a reference for future large-scale genome engineering work in this and other similar recombinant protein production hosts.

## 1. Introduction

Cell lines derived from *Trichoplusia ni*, the cabbage looper moth, have been used for many years to produce recombinant proteins by means of the baculovirus expression vector system (BEVS). While cell lines from other lepidopteran hosts such as *Spodoptera frugiperda* have commonly been used for production of baculoviruses, *Trichoplusia* cell lines have been shown in several cases to out-perform these cell lines for production yield and protein quality, particularly with regard to secreted proteins [1]. In the past decade, insect cell protein production has emerged as a viable alternative to bacterial and mammalian cells for the production of therapeutically relevant proteins, with several vaccine products generated in baculovirus-infected insect cells having been approved by regulatory agencies [2,3]. Therefore, a comprehensive systems biology approach to improving protein production in these cell lines would be of significant benefit to their potential utility as protein production hosts. However, to date, there are only a few complete genome sequences of lepidopteran hosts. One of them is the silkworm *Bombyx mori*, that has been published [4], while an incomplete draft genome of *Spodoptera frugiperda* (the host from which Sf9 and Sf21 lines were derived) is the only sequence available for the more commonly used protein production hosts [5]. The transcriptome [6] of the *Trichoplusia ni*-derived cell line, Tnms42, and RNA-seq data [7] from the High Five cell line (BTI-Tn-5B1-4), have been published, but these data are not useful for large-scale genome engineering due to a lack of non-coding genomic DNA information. In addition, transcriptome data are inherently biased towards genes with high transcription levels, and likely lack coverage of significant regions of the coding genome.

Tni-FNL is a cell line derived by adaptation of BTI-Tn-5B1-4 cells, originally isolated from *Trichoplusia ni* egg cells in the Wood laboratory at Cornell [8]. While the original cell line was an adherent cell line that grew in the presence of serum, Tni-FNL was selected for suspension growth to optimize its utility for protein production and for the ability to grow in the absence of serum. The Tni-FNL cell line has been shown to routinely produce higher levels of protein than Sf9 or Sf21 cells, and in some cases to surpass the levels produced in the more commonly used and commercially available *Trichoplusia ni* cell line, High Five. High Five cells were derived from the same parent line as Tni-FNL, suggesting that the specific process used for adaptation likely effected changes in the cell line, which resulted in this improvement in protein production. For these reasons, we decided to elucidate the complete genome sequence of the Tni-FNL cell line. This will benefit systems biology approaches to create improved cell lines that can support higher levels of protein expression and potentially improve the quality and lower the cost of therapeutic protein production.

Next-generation sequencing technologies have long been used in the genome assembly of many animal and plant genomes. However, the short-reads they produce have difficulty spanning repetitive regions commonly found in many genomes, and therefore, generate draft genomes consisting of many gaps with potential mis-assemblies and collapsed contigs. Recent advances in sequencing technologies, especially in single-molecule sequencing [9], have resulted in the ability to sequence reads that are longer than most of the common repeats in both microbial and vertebrate genomes, leading to the generation of highly contiguous assemblies. Combining PacBio single-molecule sequencing [9] with complementary technologies such as Illumina short reads, Bionano optical mapping [10], and 10X Genomics (Pleasanton, CA, USA) linked reads [11] has become the recommended strategy for optimal genome assembly [12]. Here we report that by applying the new technologies and assembly strategies, we have generated the first draft genome assembly of Tni-FNL cell line, which was derived from *Trichoplusia ni* cells. Comparative analysis between our draft genome of the Tni-FNL (*Trichoplusia ni*) genome with other closely related species, as well as the recently published Hi5 germ cell genome assembly [13], provided further evidence of high accuracy and completeness of our Tni-FNL cell line genome assembly.

## 2. Materials and Methods

### 2.1. Cell Culture Conditions

Tni-FNL cells were cultured under shaking conditions (125 rpm, 2-inch throw) in 500 mL shaker flasks at 27 °C in Gibco Sf-900 III SFM media (Thermo Fisher Scientific, Waltham, MA, USA).

### 2.2. PacBio Library Preparation and Sequencing

High-molecular-weight genomic DNA (20–150 kb) was extracted from the cultured Tni-FNL cell line using the Genomic-tip 20/G kit (Qiagen, Hilden, Germany). For PacBio library preparation, approximately 15 µg of genomic DNA were sheared to an average size of 20 kb using a g-TUBE™ (Covaris^®^, Woburn, MA, USA). All sizing and quantitation measurements were performed using the genomic kit for the TapeStation 2200 (Agilent Technologies, Santa Clara, CA, USA). Purity was assessed by calculating the ratio of absorbance at 260 nm to absorbance at 280 nm as measured on a NanoDrop™ spectrophotometer (Thermo Fisher Scientific, Waltham, MA, USA) and was determined to be suitable. Following PacBio’s standard 20 kb library preparation protocol 100-286-000-05, the final library was size selected using a dye-free 0.75% agarose cassette on a BluePippin (Sage Science, Beverly, MA, USA) with a lower cutoff of 10 kb. 16 SMRT^®^ cells were sequenced on the PacBio RS II (Pacific Biosciences, Menlo Park, CA, USA) using P6/C4 chemistry, 0.15 nM MagBead loading concentration, and 360 min movie lengths. Additionally, 5 µg of genomic DNA from the same sample were sheared to an average size of 20 kb using a g-TUBE (Covaris, Woburn, MA, USA), which was used as input to create a library using the Accel-NGS^®^ XL Library Kit for Pacific Biosciences^®^ (Swift Biosciences™, Ann Arbor, MI, USA). The final library was size selected using a dye-free 0.75% agarose cassette on a BluePippin (Sage Science, Beverly, MA, USA) with a lower cutoff of 15 kb. 2 SMRT^®^ cells were sequenced on the PacBio RS II (Pacific Biosciences, Menlo Park, CA, USA) using P6/C4 chemistry, 0.15 nM MagBead loading concentration, and 360 min movie lengths. Additionally, approximately 15 µg of genomic DNA were sheared to an average size of 20 kb and were prepared for sequencing on the PacBio Sequel System, using a size selection with a 15 kb lower cutoff on the BluePippin. Two Sequel 1M SMRT^®^ Cells were sequenced using Sequel Polymerase 2.0 and Sequel Sequencing Kit 2.0 (Pacific Biosciences, Menlo Park, CA, USA).

### 2.3. Bionano Optical Mapping

Optical mapping was performed using the Irys optical mapping technology from Bionano Genomics (San Diego, CA, USA). The sample was prepared as per the IrysPrep Plug Lysis protocol 30026 Rev D and Labeling-NLRS protocol 30024 Rev J. Two million cells from the Tni-FNL cell line were embedded in an agarose plug for extraction of ultra-high-molecular-weight genomic DNA (100–2000 kb). Briefly, the cells were washed with phosphate buffered saline (PBS), the cell suspension was mixed thoroughly with 2% Agarose, and then set into cold plug molds for 15 min. Plugs were treated overnight with Proteinase K at 50 °C, followed by RNase A digestion at 37 °C for 1 h. After washing the plugs with wash buffer and TE, DNA were recovered by incubating the molten plug with Agarase for 45 min at 43 °C. The DNA were further cleaned by Drop Dialysis using a 0.1 µm dialysis membrane set on top of TE in a petri dish. The DNA were dispensed on top of the membrane and dialyzed for 45 min. Homogenization of the DNA was achieved by overnight incubation at room temperature. 600 ng of purified high molecular weight DNA were nicked using 80 Units of the nicking endonuclease Nb.BssSI (New England Biolabs, Ipswich, MA, USA) for 2 h at 37 °C. Fluorescently tagged nucleotides were then incorporated at the nicked sites by Taq DNA polymerase during the labeling reaction at 72 °C for 60 min. This was followed by repair in the presence of polymerase and Taq DNA ligase for 30 min at 37 °C. After counterstaining the DNA backbone with the YOYO-1 dye, the final sample was quantitated again and 9 µL were loaded into each flowcell of an IrysChip. The labeled DNA molecules were linearized in the nanochannels on the chip and imaged by the Irys instrument (Bionano Genomics, San Diego, CA, USA). Both flowcells were run per the Modified Base Recipe for 30 cycles, with the DNA concentration time of 200 s. After the first run, pillar cleaning of the chip was performed, and the chip was imaged again for an additional 30 cycles. This over-cycling was performed three additional times for both flowcells of the IrysChip (30 cycles in each run) to acquire additional data.

### 2.4. 10X Genomics Linked Reads Sequencing

High-molecular weight DNA from the Hi5 cells (extracted using the Bionano Plug Lysis protocol) was also used to make 10X libraries, as per the Chromium Genome library preparation protocol from 10X Genomics (Pleasanton, CA, USA). In brief, 0.9 ng/µL DNA were used for GEM generation in the Chromium Controller machine (10X Genomics, Pleasanton, CA, USA). The long DNA molecules were partitioned along with oligo-coated Gel Beads that provide a 16 bp 10X barcode, an Illumina R1 sequence, and a 6 bp random primer sequence. Isothermal incubation of the GEMs at 30 °C for 3 h, followed by 65 °C for 10 min produced barcoded fragments. These fragments were recovered from the GEMs and cleaned up for subsequent library preparation steps that included end repair, A-tailing and adapter ligation per the manufacturer’s recommendations. Eight cycles of amplification during the sample index PCR provided enough yield of the indexed library. The library was quantitated by qPCR and sequenced on NextSeq (High output kit) (Illumina, San Diego, CA, USA) with 2 × 150 paired-end reads.

### 2.5. Transcriptome Sequencing of Tni-FNL Cell Line

Total RNA was extracted from Tni-FNL cells using the NEB Monarch Total RNA Miniprep kit (New England Biolabs, Ipswich, MA, USA) as per the manufacturer’s instructions. Briefly, a frozen pellet of approximately 20 million cells was thawed and resuspended in RNA lysis buffer. Genomic DNA was removed by binding to a gDNA removal column, followed by purification of RNA by binding to the RNA purification column. On-column DNase I treatment was performed for removal of residual gDNA. RNA was eluted in nuclease-free water and RNA integrity was assessed using the RNA 6000 Nano Kit on an Agilent 2100 Bioanalyzer (Agilent Technologies, Santa Clara, CA, USA). Approximately 20 µg RNA was obtained and aliquoted before storage and further use.

Short-read sequencing library was prepared from the Tni-FNL total RNA using the NEBNext Ultra II Directional RNA Library Prep kit (New England Biolabs, Ipswich, MA, USA) as per the manufacturer’s instructions. Briefly, 1 µg and 500 ng total RNA was subjected to rRNA depletion by rRNA probe hybridization and RNase H digestion. The excess probes were removed by DNase I digestion and the RNA purified using RNAClean XP beads (Beckman Coulter, Brea, CA, USA). RNA was fragmented at 94 °C for 5 min to generate a large insert library. Accordingly, longer incubation time (50 min at 42 °C) was used during first strand cDNA synthesis. Purified double-stranded cDNA was subjected to end prep and adaptor ligation as per the protocol. After purification and selection for larger size fragments, the adaptor ligated DNA was enriched using 9 cycles of PCR amplification and purified using SPRIselect beads (Beckman Coulter, Brea, CA, USA). Library quality assessment on Agilent 2100 Bioanalyzer (Agilent Technologies, Santa Clara, CA, USA) revealed the average library sizes to be around 430 bp.

Total RNA libraries generated from 1 µg and 500 ng Tni-FNL RNA were pooled and quantified by qPCR. Paired-end sequencing (150 bp reads) was performed on the Illumina (San Diego, CA, USA) MiSeq platform using v2 sequencing chemistry.

### 2.6. Propidium Iodide (PI) Staining for Ploidy Determination

Tni-FNL and Sf9 cells in exponential growth phase were harvested at a cell concentration of approximately 1 × 10^6^ cells/mL and centrifuged at 1050 rpm for 5 min after which the supernatant was discarded. The cell pellet was suspended in 20 mL of cold (−20 °C) 70% ethanol for fixation and the samples were stored at −20 °C. On the day of flow cytometry analysis, cell samples were centrifuged at 1050 rpm for 5 min to remove the ethanol fixative. The supernatant was discarded and the cells were washed two times with 10 mL phosphate buffered saline (PBS). The sample was centrifuged again at 1050 rpm for 5 min, the supernatant was discarded, and the pellet was suspended in 1 mL of RNase solution (250 μg/mL; Sigma, St. Louis, MO, USA) for 20 min at 37 °C. A 50 μL aliquot of propidium iodide (PI; 50 μg/mL) was added to each sample, mixed, and incubated at room temperature for 5 min, before analysis by flow cytometer.

### 2.7. Flow Cytometry Analysis

Flow cytometric analysis was performed using the LSRFortessa (BD Biosciences, San Jose, CA, USA) SSC-A vs FSC-A with a gate for cell population. Single cells were selected for analysis by using the distribution of propidium iodide-W against propidium iodide-A to discriminate doublets and debris. The propidium iodide-A voltage was adjusted to set the mean of the singlet peak of the Sf9 cell (reference cell) G0/G1 population at 50,000 in the histogram. The data were collected using FACSDiva software version 8.0 (BD Biosciences, San Jose, CA, USA) and analyzed by FlowJo software version 10.2 (FlowJo, Ashland, OR, USA). The DNA index was calculated as the ratio of the mean fluorescence intensity (MFI) of the Tni-FNL cell G0/G1 population to the MFI of the normal reference (Sf9) G0/G1 population. Ploidy of the test sample was then calculated based on the DNA index and the ploidy of the normal reference.

### 2.8. Karyotype Analysis

Chromosome preparations were obtained from established cultures of Tni-FNL. Vinblastine (5 mg/mL; Sigma, St. Louis, MO, USA) was added to the cells for 2 h prior to harvest and incubated at 27 °C. Cells were treated with hypotonic solution (KCL 0.075M) for 20 min at 37 °C and fixed with methanol: acetic acid 3:1. Slides were prepared at 60% humidity and aged overnight. Pairing was completed using slides that were stained with a trypsin-Giemsa staining technique (GTG). Analyses were performed under an Axio Imager Z2 (Zeiss, Oberkochen, Germany) microscope coupled with a VDS CCD-1300 camera (Genasis, ASI, Carlsbad, CA, USA); images were captured with Spectral Acquisition Band View 7.2 karyotyping software, (Applied Spectral Imaging Inc., Carlsbad, CA, USA).

### 2.9. NGS De Novo Assembly Methods

The genomic libraries were sequenced on two different sequencing platforms including the PacBio Sequel and RSII systems (Pacific Biosciences, Menlo Park, CA, USA) and the Illumina NextSeq 500 (Illumina, San Diego, CA, USA). We performed de novo assembly of the PacBio sequencing reads using the HGAP4 assembler [14] from SMRT Link software version 4.0.0 (Pacific Biosciences, Menlo Park, CA, USA). The HGAP4 assembly consensus was polished using the Quiver software in the SMRT Link software package. In addition, the Canu v1.4 assembler [15] was used to generate a second set of primary assembly. The Canu assembler was run with all three options of trimming, error correction and assembly. 10X Genomics Supernova v1.2.0 (10X Genomics, Pleasanton, CA, USA) was run iteratively for subsampling in order to find the best genome coverage and optimal assembly results. We subsampled 42× linked reads sequence data and performed de novo assembly.

### 2.10. Bionano De Novo Assembly

De novo assembly was done using the Bionano Genomics RefAligner version 5122 software (Bionano Genomics, San Diego, CA, USA). First, we merged all the Bionano runs using the merge function of the IrysView. Then the merged molecules set was used for Bionano de novo assembly. The converted Hierarchical Genome Assembly Process (HGAP4) assembly Consensus Map (CMAP) file was also supplied for the error rate estimation. Analysis parameters were given from the optArguments_human.xml. We generated multiple assemblies using the different minimum length cutoffs (150 kb, 180 kb and 210 kb) with two different CMAP-converted fasta assemblies (HGAP4 and Canu). After checking the resulting de novo assemblies, we decided to use the 150 kb minimum length cutoff in conjunction with the HGAP4 fasta file supplied as the CMAP file for our final de novo assembly.

### 2.11. Bionano Hybrid Assembly

For the step-one hybrid assembly (V1) we used the de novo Bionano assembly with the HGAP4 fasta assembly using the parameters from the aggressive human assembly setting, choosing Nb.BssSI (New England Biolabs, Ipswich, MA, USA) as the enzyme and a threshold *p* value of 1 × 10^−10^. In the two steps of hybrid scaffolding to align Bionano genome maps with PacBio WGS assemblies, the parameters -B2 and -N1 were used to only cut optical mapping assemblies when a conflict was found. For the step-two (V2) version of the hybrid assembly, we merged the mapped CMAP file in the hybrid V1 assembly with the unmapped CMAP file not used in the hybrid assembly with RefAligner version 5122 (Bionano Genomics, San Diego, CA, USA). Then we used the merged CMAP from V1 hybrid with the Canu assembly to carry out the V2 version of the hybrid assembly. The same parameters were used for the V2 hybrid assembly.

### 2.12. Assembly Error Correction

The final hybrid scaffold assemblies were error-corrected using Pilon [16]. The raw Canu assembly was mapped to Illumina data using the BWA-mem aligner. After mapping, the bam file and Canu raw assembly were supplied to Pilon to perform error correction.

### 2.13. Transcriptome Assembly

rCorrector [17] was used to remove erroneous k-mers from Illumina paired-end short reads. Adapters and low-quality reads were trimmed using trimmomatic tool. The trimmed pair-end reads were assembled by using trinity assembler (--SS_lib_type FR and --min_kmer_cov 1). The assembly statistics was calculated using Quast. The completeness of the assembly was assessed using BUSCO against Endopterygota database.

### 2.14. Gene Predictions and Repeat Annotations

The complete genes were predicted from the repeat masked genome using Maker v 2.31.8 pipeline [18] as described in the GC Specific Maker pipeline. After the first initial run of Maker with est2genome [19], the resulting annotation was divided based on GC content as high and low GC data sets. High and low GC datasets along with the original first maker annotations were used to train the SNAP [20] and Augustus [21] HMMs for the gene prediction. In the final run, the assembly was trained against six models, including three from SNAP and three from Augusts, using Maker. The high quality gene models were filtered by choosing Annotation Edit Distance (AED) cut off 0.5 according to the published Maker protocol [22].

### 2.15. Phylogeny Analysis for Ten Insect Genomes

Genomes of *Bombyx mori* (GCA_000151625.), *Cimex lectularius* (GCA_001460545.1), *Bombus terrestris* (GCF_000214255.1), *Bombus impatiens* (GCF_000188095.1), *Helicoverpa zea* (GCA_002150865.1), *Mamestra configurata* (GCA_002192655.1), *Helicoverpa armigera* (GCA_002156985.1) and *Cimex lectularius* (GCA_000648675.1). were downloaded from NCBI. For *Drosophila*, the BDGP6 version of the genome was used. Nine genomes described above and the *Trichoplusia ni* Tni-FNL assembly were used with Busco [23] to annotate the completeness of single-copy orthologs. We used a total of 250 strict one-to-one orthologs from the 10 species to run the phylogeny analysis. One fasta sequence was generated per species by appending each of the 250 ortholog sequences. The final file containing a single sequence per species was used for multiple sequence alignment by MUSCLE [24]. RAXML [25] was used to generate the maximum likelihood phylogeny from the concatenated multiple sequence alignment using 1000 bootstrap. The resulted Newick formatted tree was plotted using the iTOL [26].

## 3. Results

### 3.1. Genome Sequencing and Assembly

To build the assembly, we used a combination of three technologies, including PacBio single-molecule long-read sequencing, Bionano optical genome mapping, and 10X Genomics long linked-reads. For PacBio sequencing, we constructed and sequenced a 20 kb SMRTbell library using 16 SMRT cells on the PacBio RS II. 27.3 Gb of data were generated with a 7.2 kb mean subread length. An additional 20 kb library was constructed using the Swift Biosciences Accel-NGS^®^ XL Library Kit (Swift Biosciences™, Ann Arbor, MI, USA) and was sequenced using 2 SMRT cells on the PacBio RS II, generating 3.1 Gb of data with a 9.7 kb mean subread length. In addition, a 20 kb library was prepared by PacBio and sequenced using 2 SMRT cells on the PacBio Sequel platform, generating an additional 11 Gb of data with a 11 kb mean subread length. In total, 4,236,403 subreads composing 41 Gb were produced by the PacBio platforms, representing approximately 110× coverage of the genome (Table S1). We first performed a contamination check of the PacBio long reads using DeconSeq [27] and found no contaminants in the sequencing reads. We then performed de novo assembly of the PacBio long reads using both the HGAP4 assembler [14] and the Canu v1.4 assembler [15]. The HGAP4 assembly was error corrected and polished using Quiver in the SMRT Link software package. The resulting HGAP4 assembly had 1428 contigs with a total length of 366.3 Mb. The HGAP4 contig N50 size was 939.8 kb and the maximum contig size was 4.35 Mb. The Canu assembly was performed using the options of trimming and error correction and yielded 2101 contigs, with a total size of 408.4 Mb. The Canu N50 contig size was 737.2 kb and the maximum contig size was 6.1 Mb. Since the HGAP4 and Canu v1.4 assemblers utilize different algorithms, the results may differ and be complementary, potentially providing better coverage of the whole genome when both are considered during downstream analysis.

The 10X Genomics linked-reads library was constructed and then sequenced on an Illumina NextSeq 500, producing 918 million 2 × 150 pair-end reads, with an estimated 338× depth of coverage. We ran the 10X Genomics Supernova version 1.2 (10X Genomics, Pleasanton, CA, USA) de novo assembly software [28] and sub-sampled the barcoded reads to get an effective coverage of approximately 42× of the whole genome for an optimal assembly result. The Supernova total scaffold size was 375.8 Mb with a maximum scaffold of 7.83 Mb and N50 size of 1.63 Mb. The Supernova total contig size of 316.7 Mb was comprised of 18,923 contigs with a N50 contig size of 54 kb including 6467 contigs exceeding 10 kb in length. The Supernova assembler produced a much more fragmented assembly, with a total contig size much shorter than that of the PacBio WGS assemblies produced by the HGAP and Canu v1.4 assemblers. Table 1 shows the comparative assembly metrics generated by using QUAST [29] for the three NGS assemblies.

### 3.2. Improved Genome Assembly Using Bionano Optical Mapping Data

**Figure 1:**
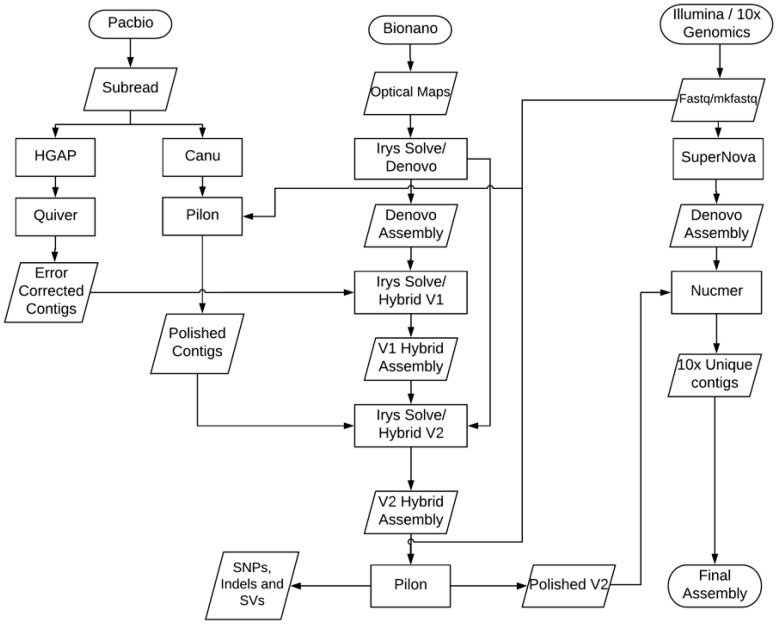
Genome assembly and optical maps hybrid scaffold workflow. The workflow steps for de novo assembly, hybrid scaffold, genome assembly error correction and polishing.

To improve the PacBio assemblies and generate hybrid scaffolds representing chromosomal structure, we used Bionano’s Irys System to generate optical mapping data. Bionano maps can order and orient sequence fragments to build scaffolds, identify potential chimeric joins in the sequence assembly, and resolve conflicts between a WGS assembly and genome maps [30]. The high-molecular-weight (HMW) DNA molecules were nicked using Nb.BssSI enzyme (New England Biolabs, Ipswich, MA, USA), based on an optimal label density of 14.1 labels per 100 kb, as predicted by the Knickers software (Bionano Genomics, San Diego, CA, USA) from Bionano. We ran this library on the Irys system on 6 flowcells, and merged all 6 runs of molecule data for de novo assembly. The resulting de novo assembly of maps had an average depth of molecule coverage of 61.8× and a total size of 645 Mb in 1272 Bionano maps. Among these 1272 maps, 730 were between 10–500 kb, 469 were between 500–1000 kb, 71 were above 1000 kb and 2 were longer than 2000 kb. The N50 map size was 608 kb. We chose maps greater than 150 kb and combined them with the PacBio WGS assemblies to produce ultra-long hybrid scaffolds using a two-step hybrid approach (Figure 1). We used the Bionano Hybrid Scaffold pipeline (Bionano Genomics, San Diego, CA, USA) [30] to produce “V1” hybrid scaffolds with the HGAP4 WGS assemblies. A total of 301 V1 hybrid scaffolds were produced, with a total assembly size of 328.2 Mb. The N50 of the resulting assembly was 1.74 Mb and the maximum scaffold size was 10.4 Mb. The Bionano software produces a CMAP file as output, which is a raw data view of a molecule set or assembly reporting the label site positions within a genome map. We then merged the mapped CMAP file from the V1 hybrid assembly with the unmapped CMAP files and used Canu v1.4 assembly to carry out a “V2” hybrid assembly. The V2 scaffold reduced the total number of scaffolds from 301 to 280, further increased the N50 size to 2.33 Mb and produced a longer maximum scaffold size of over 12.8 Mb. The total assembled scaffold size increased to 359.1 Mb. This V2 hybrid assembly significantly improved the scaffold N50 size by two-fold compared to the HGAP4 WGS assembly. Additionally, the longest scaffold size in the V2 hybrid assembly is twice the length of the longest contig size in the HGAP4 WGS assembly (Table 2). The gaps (fraction of Ns) in the V2 scaffolds were only 5.5% of the total 359.1 Mb scaffold size.

### 3.3. Assembly Conflict Resolution

We identified 789 inconsistent regions when comparing the PacBio WGS assemblies to the Bionano genome maps. We specifically looked for chimeric joins, which are formed when PacBio reads are too short to span across extremely long DNA repeats. These errors would appear as conflicting junctions in the alignment between the PacBio WGS assemblies and Bionano genome maps [30]. The Bionano de novo assembly software reported the conflict regions as alternative consensus maps representing different haplotypes. There was a total of 1029 WGS contigs, of which 637 (61.9% of the total) anchored within the hybrid scaffolds when using a *p* value of 1 × 10^−10^ as the cutoff threshold. Among the 789 identified conflicts, 199 chimeric junctions were identified and automatically resolved by the Irys hybrid scaffold software (Figure S1). The high number of remaining unresolved conflicts between the WGS assemblies and Bionano optical maps, as well as a high abundance of short fragment sizes composing the optical maps, suggests the presence of shorter molecules in the Bionano library, and also indicates that the Tni-FNL cell line genome may be highly polymorphic. It was previously reported that insect cell lines used to produce recombinant proteins are cytologically unstable, resulting in varying numbers of chromosomes, depending on the culture history and supplier [31].

### 3.4. Error Correction of Genome Assembly

The final assembled genome sequences were error-corrected by using two software tools: SMRT analysis resequencing module and Pilon pipeline software [16]. We first mapped the PacBio quality-filtered reads to the hybrid assembly sequences to identify consensus and variant sequences using the PacBio Quiver software. This produced both BAM files and lists of variants in VCF format.

Pilon was used to improve the final hybrid assembly by using read alignments from the Illumina 10X Genomics linked-read data set. Pilon found and fixed 1566 SNPs, 5996 small insertions (consisting of 8848 bases), 11 small deletions (consisting of 3237 bases), 1164 local misassembles, and 2 gaps. This step reduced the total gap size by 26.8 kb in the final hybrid scaffold, and produced a polished final draft genome assembly (Table 2). Of the final 280 scaffolds, 171 scaffolds have sizes greater than 500 kb, 4 scaffolds have sizes less than 100 kb, and the rest of the scaffolds have sizes between 100 kb and 500 kb. (Figure S2).

### 3.5. Genome Size Estimation Based on K-Mers

To estimate the genome size independently of assembly, we characterized the genome sequence using k-mer histograms, which was computed from the error-corrected reads using the program Jellyfish [32], with word sizes k from 19 to 31. Figure S3 shows the k-mer plot of 1N genome. We also used GenomeScope [33] to profile the genome from the complete set of Illumina short reads. This method gave an estimated haploid genome length of about 328 Mb, and estimated 86.2% of the genome was unique and the overall rate of heterozygosity of the genome was 0.35%, based on k-mer 27 profiling. The lower heterozygosity of the Tni-FNL cell line genome suggests that the Tni-FNL cells were relatively homogenous. This observation is consistent with the recently published Hi5 cells genome sequencing paper, which concluded that the Hi5 cells originated from a single founder cell or a population of homogenous cells, which are different from animal genome.

### 3.6. Genome Assembly Quality and Completeness Assessment

We first mapped the Illumina 10X Genomics pair-end reads to our Tni-FNL draft genome sequence. Of the total 918.4 million reads, 93.6% was mapped to the 359 Mb draft genome sequence. Only 0.05% of the draft genome had no sequencing coverage from Illumina pair-end reads. This indicates that our Tni-FNL genome assembly is nearly complete.

In addition, we mapped the 10X Genomics Supernova contigs to our Tni-FNL draft genome assembly sequences using Nucmer [34] and produced a mapped BAM file. Of the total 276.35 Mb 10X Genomics Supernova contigs that were longer than 10 kb, only 7.12 Mb were either totally unmapped or partially unmapped, composing only 2.9% of the 10X Genomics Supernova contigs that were not represented in the draft Tni-FNL genome assembly. From the set of 10X Genomics Supernova contigs that were aligned with the draft genome assembly, 99.4% were also aligned concordantly (Figure S4), indicating that the assembly was correct at the local level. Comparing the 10X Genomics Supernova de novo assembly with the Bionano consensus genome map and WGS hybrid assembly, the latter had a much longer N50 scaffold size and better contiguity.

We also compared the transcriptome data of the High Five cell line published in 2016 [7] to our Tni-FNL draft genome assembly. Among the 25,234 assembled transcripts in the High Five cell line, 95.1% were mapped to our Tni-FNL draft genome assembly and 91% were uniquely mapped. This further indicates that our draft genome assembly is correct at the local level and nearly complete.

Compared with the two other lepidopteran genome assemblies, *S. Frugiperda* (358 Mb) [5] and *B. mori* (432 Mb) [4], our genome assembly of the *Trichoplusia ni*-derived Tni-FNL cell line produced much longer N50 contig sizes and far fewer gaps, representing the most contiguous genome assembly for any lepidopteran genome to date (Table 3).

### 3.7. Determination of Cell Line Ploidy and Karyotype Analysis

Previous analysis of the chromosome content of lepidopteran cell lines showed a lack of consistency in the ploidy of these cells. Sf9 cell lines were shown to have a mixed population of diploid and tetraploid cells that varied in their ratios even when cells were cloned out [35]. *Bombyx mori* cell lines were mostly diploid but by examining a number of lepidopteran lines, ploidy was found to be highly variable [36]. While no characterization of T. ni cell line ploidy has been published so far, based on cell size data, we speculated that it was very probable that these cell lines were tetraploid. Propidium iodide staining confirmed most Tni-FNL cells contain a 4n DNA content (Figure S5), similar to the profiles previously observed for selected tetraploid Sf9 cells. In contrast, our Sf9 control cell line (derived from ATCC CRL-1711) is mostly diploid.

Lepidopteran chromosomes have been shown to be holokinetic, lacking centromeric structures, which makes them highly prone to chromosome fragmentation and complex karyotyping [37,38]. Due to this lack of centromeres, different karyotypic staining techniques were tried. In the end, pairing was completed using slides that were stained with a trypsin-Giemsa staining technique (GTG). The GTG banding provided slight differences in the structure of the chromosomes that were used to match similar chromosomes and fragments. As shown in Figure S6, karyotyping of the Tni-FNL cells was consistent with the suggestion of a tetraploid genome with visible chromosome fragmentation. The average chromosome number over several spreads was 130. In the context of the putative tetraploid state of Tni-FNL, these data are consistent with the previous identification of 28–30 chromosomes for most lepidopteran organisms. The chromosome number appears to be lower than previous studies on Sf9 cells, which suggests much higher levels of chromosome fragmentation in those cells [35].

### 3.8. Analysis of GC Content and CpG Islands

CpG islands, which are clusters of CpG dinucleotides in GC-rich regions, represent important features in insect genomes. Analysis of GC content and CpG islands can help identify the role of CpG islands in gene regulation and evolution [39]. A previous study found uniformity in GC content and CpG islands among the lepidopteran insects [10]. Our draft assembly of the Tni-FNL genome shows a GC composition of 35.7% of the total bases. This composition is very similar to the closely related species *S. Frugiperda* (33.0%), and *B. mori* (37.7%) (Figure S7) and further supports the notion that closely related organisms share common features at the genomic level.

To study CpG island distribution in the genome, we used EMBOSS [40], which enabled identification of 4426 CpG islands with a total length of approximately 2.8 Mb (0.8% of the genome) from a total of 280 final scaffold assemblies. Greater than 83.4% of the CpG islands were identified when using a genomic window length of 200–800 bases. By comparing the GC content distribution against the occurrence of CpG islands, we found that the CpG islands occur in the genome where the GC content ranges between 51% and 75%. The average GC content within the CpG islands is 63.6%, while the average GC content of the whole genome assembly is 35.7%. This is similar to that of the other lepidopteran insects, such as *B. mori* and *S. Frugiperda* (Figure S8). The uniformity in GC percentage among the lepidopteran insects confirms the previous observation and supports the notion that closely related insect species share similar genomic patterns [4,41].

### 3.9. Analysis of Repeat Elements Including Endogenous Viral Elements

To identify repetitive elements within the assembled genome, we ran Repeat Modeler (http://repeatmasker.org) software for de novo modeling of the Tni-FNL genome. A model library was constructed by using the Repbase [42] known repeat library. Repeat masking was accomplished using Repeat Masker with the model library generated by the Repeat Modeler. All identified repeats were annotated using Repbase classification. Our analysis revealed a total of 517,939 standard genome repetitive elements, of which 127,344 were simple repeats, 366,173 were interspersed repeats, 42,457 were retro elements including LINEs, SINEs, and LTRS, and approximately 2,986 were DNA elements. In total, 18.8% of the Tni-FNL assembly (67.5 Mb) was found to be repetitive and a major fraction of the repeats were classified as interspersed repetitive elements (Table S2).

The percentage of the Tni-FNL genome made up of repetitive elements (Figure S7) was consistent with the numbers reported for other insect genomes, including other lepidoptera such as *S. Frugiperda* (20.28%) [5] and *B. mori* (43.6%) [4], as well as other related insects such as *C. lectularius* (31.65%) [43], *B. terrestris* (14.8%) [44], *B. impatiens* (17.9%) [44], and *D. melanogaster* (20%). We also searched the Tni-FNL genome sequence for endogenous viral elements (EVEs) including endogenous retroviruses (ERVs) among mammalian genomes, and found that 3183 bases of unique EVEs elements hit the genome. Transcriptionally active EVEs have been suggested to confer protection or tolerance against related exogenous viruses [45,46].

### 3.10. Gene Prediction and Functional Annotation

We predicted 14,101 gene models in the Tni-FNL genome based on the Maker2 [18] pipeline, which utilizes known proteins, expressed-sequence tags (ESTs), or assembled transcripts to predict gene models. The High Five cell line (BTI-Tn-5B1-4) transcriptome sequencing data, containing 25,234 transcripts [7], and the *B. mori* insect annotation, containing 19,559 protein sequences [4], were downloaded from GenBank and used as input training files for the Maker pipeline for gene annotation. After the first initial run with est2genome [19], the resulting annotation data sets were provided to the Snap [20] and Augustus [21] pipelines for gene structure prediction. The initial total number of predicted gene models in Tni-FNL genome was 41,078. We filtered false positive gene models by choosing Annotation Edit Distance (AED) less than 0.5. AED is a distance measure that summarizes the congruency of each annotation with its supporting evidence according to the published Maker protocol [22]. This produced 14,101 final gene models. The total count of final gene models of Tni-FNL is similar to the total genes predicted in closely related species such as *S. Frugiperda* (11,595) and *B. mori* (16,424), *D. melanogaster* (17,746) as well as to Hi5 germ line cell (14,037).

**Figure 2:**
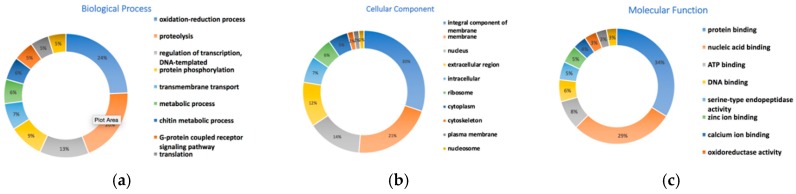
Gene ontology classification of the genes predicted. Gene Ontology (GO) classification of the predicted genes. Only the most abundance ones are displayed. (**a**) biological processes, (**b**) cellular components, (**c**) molecule functions.

To perform functional analysis of the predicted genes, we ran an InterPro [47] search against the InterPro consortium databases including Pfam, PROSITE, TIGRFAMs, CDD and 10 other databases based on homology searches. The InterPro search resulted in 13,143 protein coding genes (93.2% of total). Protein sequences were classified into families and assigned domains or functional sites. Among the 13,143 protein sequences, 8680 (66%) protein sequences were assigned Gene ontology (GO) terms, and 10,846 (82.5%) had a Pfam domain assignment. The GO terms were summarized into three main GO categories-biological processes, cellular components and molecular functions. We found a total of 884 biological processes, 316 cellular components and 952 molecular functions for the predicted gene models in the Tni-FNL genome (Table S3). The most abundant (top 10) subcategory genes are selected and shown in Figure 2.

In order to assess the quality of the genome assembly, we used a protocol developed by the Maker authors, who state that if 90% of the annotations have an annotated estimated distance (AED) less than 0.5 and more than 50% of the proteome contains a recognizable protein domain, then the genome can be defined as well annotated [22]. Our genome assembly predicts for 13,143 protein sequences that have recognizable domains or protein families assigned, which makes up 93.2% of the total proteome. The full set of 14,101 gene models all have an annotated estimated distance (AED) less than 0.5. These data strongly indicate that this genome is well annotated. It is worth noting that by incorporating RNA-seq data, as well as the annotated silkworm *B. mori* assembly [4] in our training data set, we have created an expansive gene model set, which we believe produced a more complete set of gene annotations for the *Trichoplusia ni* genome.

**Figure 3:**
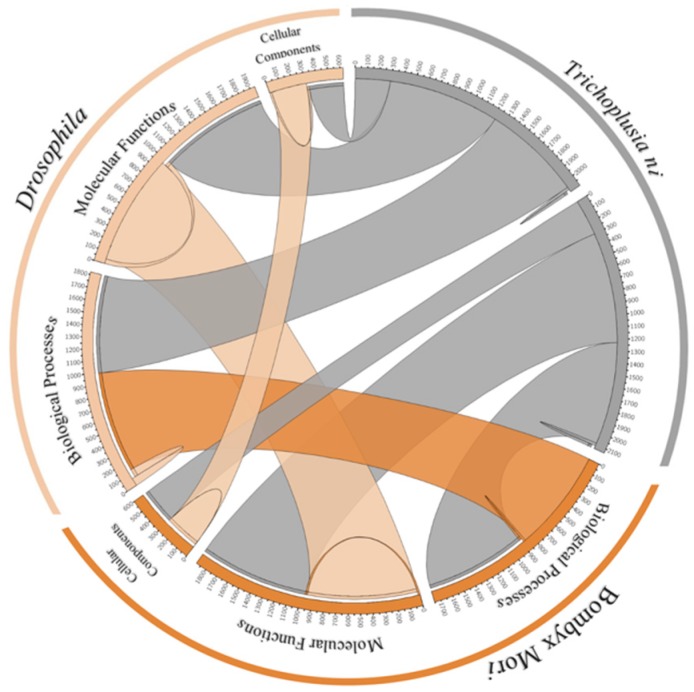
Functional annotation of the Tni-FNL (*Trichoplusia ni*), *B. mori* and *D. melanogaster* results comparison. The circos plot describes the shared cellular components, molecular functions and biological processes among the three species.

In a comparison of GO category genes of the *T. ni* genome with *B. mori* and *D. melanogaster*, the majority of these are consistent among the three species (Figure 3, Table S4). This result is consistent with earlier findings that insects share a common set of genes to maintain their integrity though their evolutionary pattern, while there is a selection for a set of genes or protein families among them conferring uniqueness to each insect [4].

We also compared the predicted gene structures of Tni-FNL with closely related species such as *S. frugiperda* (11,595 predicted genes) and *B. mori* (16,424 predicted genes). The total number of exons in the predicted genes of Tni-FNL (105,550) were between that of *S. frugiperda* (64,725) and *B. mori* (197,632). The number of exons per transcript is very similar among the three species (5.6, 7 and 8 respectively among *T.ni*, *S.f.* and *B. mori*) (Table S5). Approximately one-third of the Tni-FNL genome is comprised of genic regions and 6% by coding sequences. This is consistent with the well annotated *B. mori* annotation published in 2017 by NCBI (Annotation release 102), which shows that approximately 57% of the genome is genic and 8% contains coding sequences.

### 3.11. Orthologs and Phylogenetic Analysis

**Figure 4:**
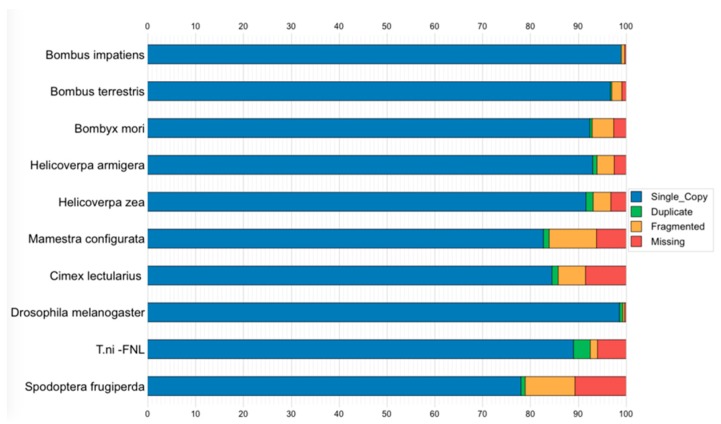
BUSCO assessment results of orthologs among 10 species. Colors refer to the percentage of the complete single-copy orthologs (blue), complete duplicated orthologs (green), fragmented or incomplete orthologs (orange), and missing orthologs (red).

For the ortholog analysis, we searched the OrthoDB6 [48] database using RefSeq genes from 10 genomes, including Tni-FNL (*Trichoplusia ni*), *Spodoptera frugiperda*, *Bombyx mori, Cimex lectularius*, *Bombus terrestris*, *Bombus impatiens*, *Helicoverpa zea*, *Helicoverpa armigera*, *Mamestra configurata*, and *Drosophila melanogaster*. BUSCO [23], a known benchmarking approach for assessing single-copy orthologs conserved among species, was used to annotate the completeness of single-copy orthologs in the above genomes. We found 2175 complete and single-copy orthologs in *Trichoplusia ni* (89.1%) out of 2442 total orthologs of the *Holometabola* lineage. This provides further evidence of high accuracy and completeness of our Tni-FNL cell line genome assembly (Figure 4).

In addition, we also found that the Tni-FNL contained a much higher number of complete and duplicated orthologs (3.5% of total 2442 orthologs of the *Holometabola* lineage) than the other 9 species in the comparison. This indicates the Tni-FNL cell line has higher levels of chromosome duplications, which suggests that Tni-FNL cells may be mostly tetraploid.

**Figure 5:**
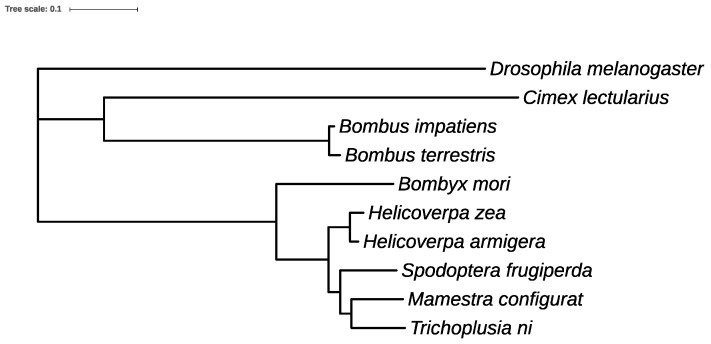
Phylogenetic analysis of *Trichoplusia ni* and closely related insect genomes. Phylogenetic analyses from 10 species including 6 lepidopterans. It depicts the relationship of *Trichoplusia ni* with the other nine insects. Maximum Likelihood tree based on a genome-wide one-to-one orthologs from 10 species. The scale bar denotes substitutions per site.

Our finding further supports previous studies showing that majority of genes have orthologous relationships across species in the same lineage, and the lineage-specific orthologs are likely to play important roles in lineage-specific biological traits [44]. In addition, we used the set of 250 strict one-to-one orthologs from all of the above 10 species for phylogenetic analysis. RAXML [25] was used to generate the maximum likelihood phylogeny from the concatenated multiple-sequence alignments. The gene content matrices were analyzed using the BINGAMMA model in RAXML. The analysis results were entirely in agreement with the accepted topology of insect relationships among the selected 10 species [26,47] (Figure 5).

## 4. Discussion

By combining PacBio single-molecule long-read sequencing with Bionano optical genome mapping and 10X Genomics long linked-reads technologies, we were able to produce a high-quality genome assembly of the Tni-FNL cell line genome. Since lepidopteran chromosomes are prone to chromosome fragmentation and complex karyotyping [37,38], assembly of a lepidopteran host genome presented a major challenge. With an average PacBio sequencing read length greater than 10 kb, the reads could easily span most repetitive elements and were unambiguously placed on the correct chromosomes, which enabled us to build a highly contiguous assembly. Two sets of WGS assemblies from the HGAP4 and Canu assemblers were generated in this process. We further improved the WGS assemblies with integration of Bionano genome maps to build hybrid scaffolds. Genome maps helped identify chimeric contigs and fixed mis-assemblies and redundancies present in the WGS contigs. We improved the hybrid scaffold results by using the long linked-reads generated from the 10X Genomics Chromium platform. The purpose of using barcoded long linked-reads that originated from larger, single molecules of DNA was to replace the approach of using costly BAC clone or pooled fosmid clone libraries. The 10X linked-reads assemblies can effectively measure the contiguity and completeness of the hybrid scaffolds produced from the PacBio and Bionano data and help to identify connection errors found in the PacBio-Bionano hybrid assembly. By combining single-molecule sequencing with complementary technologies such as optical genome mapping and 10X linked-reads, we produced a high-quality genome assembly, which comprises 359 million base pairs with fewer than 5.5% of gaps. It represents one of the most contiguous draft assemblies of a lepidopteran host genome to date.

To further assess the genome assembly quality and elucidate gene functions in this lepidopteran host, we performed the gene prediction and comparative analysis with other insect genomes, as well as utilized the transcriptome sequencing data for this cell line and data generated from previous studies [6,7]. Comparative analyses of orthologs from the Tni-FNL (*Trichoplusia ni*) genome and other lepidopteran hosts confirmed the previous study findings that insect genomes share a common set of genes to maintain their integrity through their evolution [44]. The total number of repeat elements identified in the Tni-FNL genome is very similar to those reported in other lepidopteran insect genomes. The results offer insights for further studies on how changes in the degree of repeat regions are involved in maintaining genome integrity among insert genomes.

The predicted genes of Tni-FNL identified in this study also provide additional resources for studying genetic variations and genome evolution in insect genomes. As previous studies suggested, the full-genome sequences from multiple species can complement each other by clarifying gene function and organization. In addition, this work will enable efforts to develop system biology tools to improve the utility of the Tni-FNL cell line for protein production. Recent reports have demonstrated for the first time the ability to genetically engineer *Trichoplusia* cell lines using the CRISPR/Cas9 system [49]. High-quality genome modification requires detailed genomic information to ensure high efficiency of targeted modifications and reduction in unwanted off-target effects. Using the high-quality genome sequence and newly developed engineering tools, we believe it will be possible to begin to make modifications to Tni-FNL, which will ultimately improve the quality and lower the cost of therapeutic protein production using this system.

## Figures and Tables

**Table 1:** Comparison of Tni-FNL de novo assembly statistics.

Types	HGAP Contigs	Canu Contigs	Supernova Contigs
Total contigs	1428	2101	18,923
Contigs (≥1000 bp)	1418	2101	18,196
Contigs (≥10,000 bp)	1323	2097	6467
Contigs (≥25,000 bp)	1101	1780	3592
Contigs (≥50,000 bp)	706	1041	1828
Largest contig (bp)	4,352,893	6,104,320	445,812
Total length (bp)	366,261,337	408,408,011	316,721,011
GC (%)	35.66	35.74	35.33
N50 (bp)	939,843	737,233	54,240
N75 (bp)	421,565	250,244	22,505
L50 (bp)	115	158	1653
L75 (bp)	259	399	3894

**Table 2:** Comparison of Tni-FNL genome hybrid scaffold statistics.

Types	Supernova Scaffolds	Hybrid Scaffolds (V1)	Polished Hybrid Scaffolds (V2)
Total scaffolds	12,875	301	280
Scaffolds (≥1000 bp)	12,875	301	280
Scaffolds (≥50,000 bp)	355	301	280
Largest scaffold (bp)	7,830,761	10,389,188	12,760,714
Total scaffold length (bp)	375,813,451	328,208,105	359,075,955
GC (%)	35.33	35.5	35.56
N50 (bp)	1,628,260	1,737,254	2,326,860
N75 (bp)	449,524	1,019,983	1,198,934
L50 (bp)	66	57	44
L75 (bp)	172	120	98
# N’s per 100 kb	15,724	2919	5453

**Table 3:** Comparison of Tni-FNL genome assembly with other lepidopteran genome assemblies.

Types	Tni-FNL	*Bombyx mori*	*S. frugiperda*
Total length (bp)	359,075,955	481,803,763	358,050,723
Total scaffolds	280	43,462	37,243
Ungapped length (bp)	339,494,557	431,707,935	332,569,779
Scaffold N50 (bp)	2,326,860	4,008,358	53,779
Total contigs	2,043	88,672	>49,244
Contig N50 (bp)	893,993	15,508	7,851
Largest Scaffold (bp)	12,760,714	14,496,184	641,448
Largest Contig (bp)	6,104,547	139,031	234,570
GC (%)	35.56	37.70	32.97
Gap size (bp)	19,581,398	50,095,828	25,480,944

## Supplementary Materials

The following are available online at http://www.mdpi.com/2073-4425/10/2/79/s1, Figure S1: Overview of the comparison of PacBio assemblies to Bionano genome maps, Figure S2: Hybrid assembly scaffolds size distribution, Figure S3: K-mer counts plot, Figure S4: Dot plots display alignment of 10X Genomics Supernova contigs to the final hybrid scaffolds, Figure S5: Flow cytometric analysis of DNA content of insect cell lines, Figure S6: Image and karyotype of Tni-FNL cell line, Figure S7: GC content and repeat elements comparison among three species, Figure S8: Identification of CpG islands in the Tni-FNL genome; Table S1: Data Generated from Three Different Technologies, Table S2: Repeat elements identified in the Tni-FNL genome sequence, Table S3: Gene ontology classification of the genes predicted from the Tni-FNL genome assembly, Table S4: Comparison of shared GO category genes from Tni-FNL, *B. mori* and *D. melanogaster*, Table S5: Transcript Structure Comparison between Tni-FNL, *S. frugiperda* and *B. mori*.
