# Supplementary material for: Genome Assembly and Annotation of the Trichoplusia ni Tni-FNL Insect Cell Line Enabled by Long-Read Technologies
Source: Genes (Basel). 2019 Jan 23;10(2):79. doi: 10.3390/genes10020079 (PMC6409714; doi:10.3390/genes10020079)
Supplement: Supplementary file 1 [file genes-10-00079-s001.pdf]

Supplementary Information accompanies this paper:

Supplementary Figures

Figure S1 (A): Overview of the comparison of PacBio assemblies to Bionano genome maps.

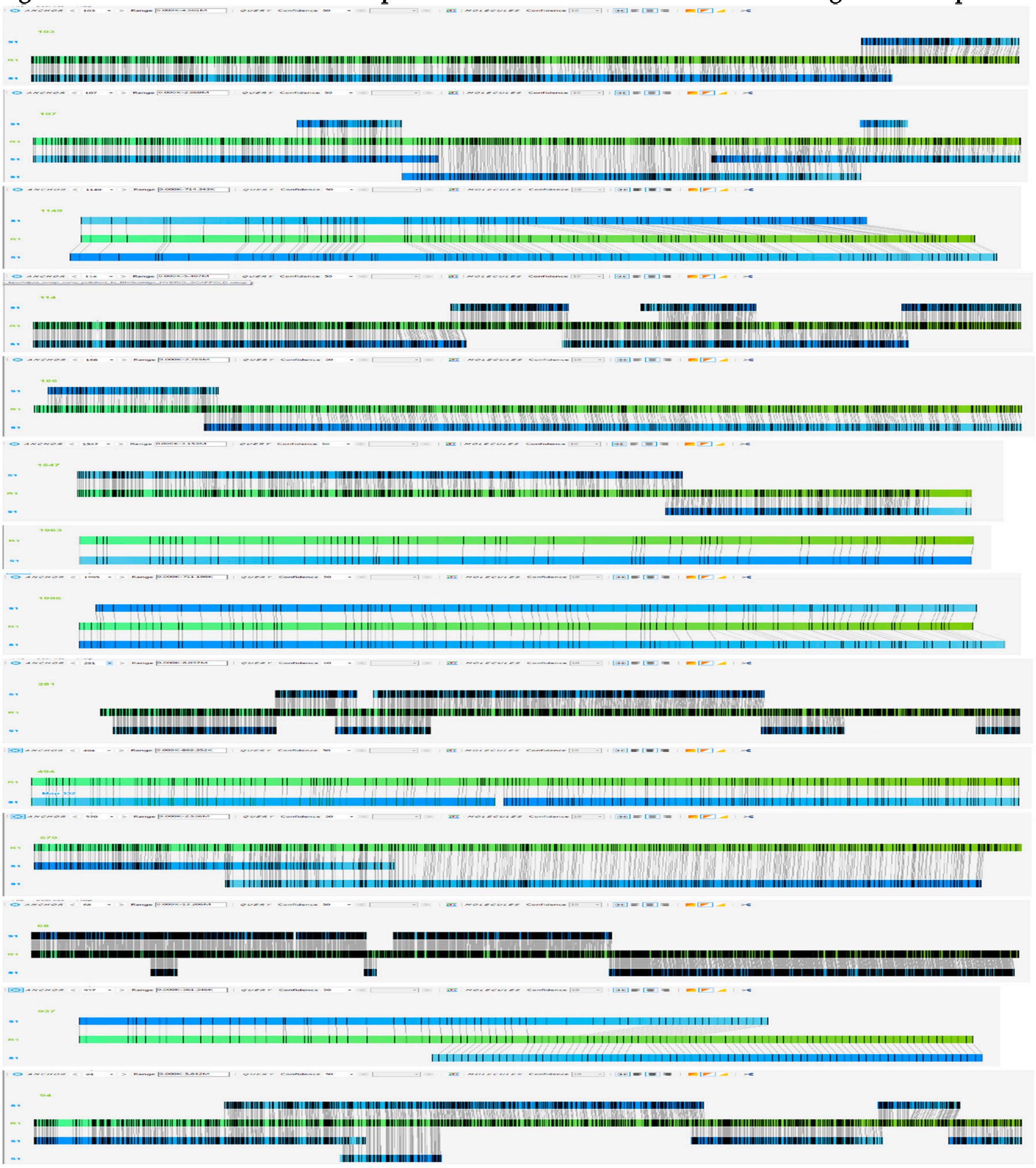

(A) The green horizontal bar represents WGS assemblies. The blue horizontal bar represents the optical maps. The vertical line represents their matching label sites.

Figure S1 (B): Overview of conflict regions of PacBio assemblies to Bionano genome maps.

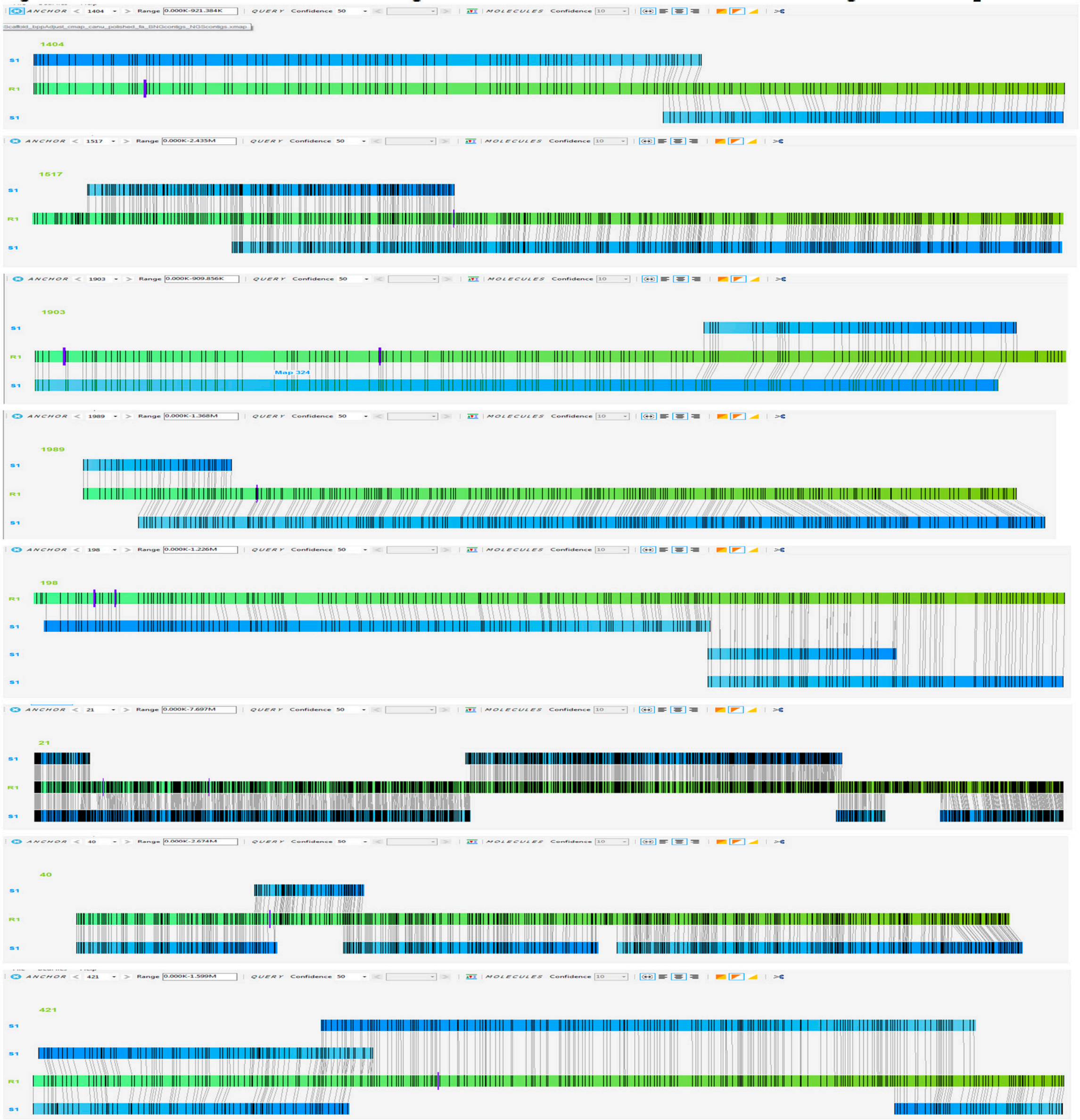

The green horizontal bar represents WGS assemblies. The blue horizontal bar represents the optical maps. The vertical line represents their matching label sites. The purple vertical bars mark where the conflicts are located on the WGS assembly when there is a conflict between WGS assembly and an optical map.

Figure S2: Hybrid assembly scaffolds size distribution.

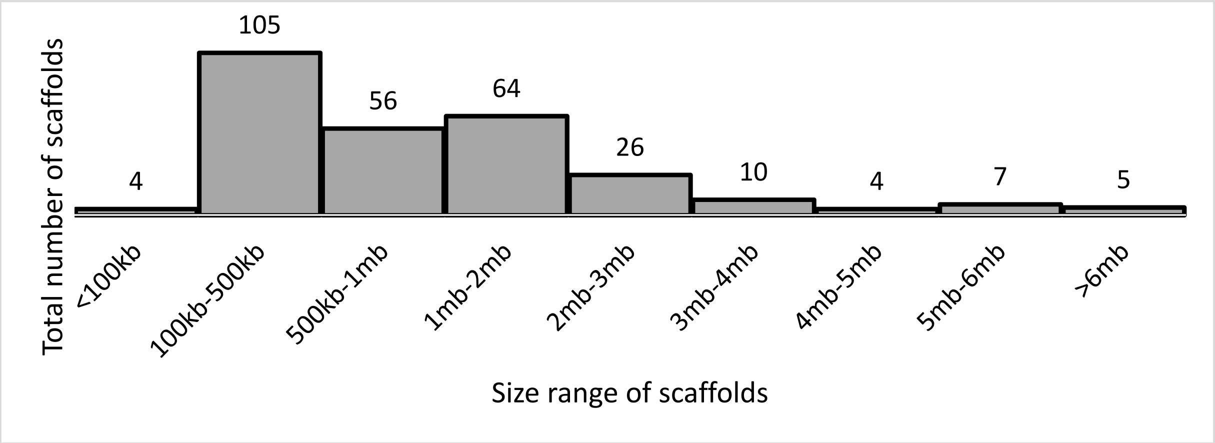

The final hybrid scaffolds are binned by different size ranges. The scaffold sizes are measured in megabase pairs (Mb), or kilobase pairs (kb), as indicated.

Figure S3. K-mer counts plot.

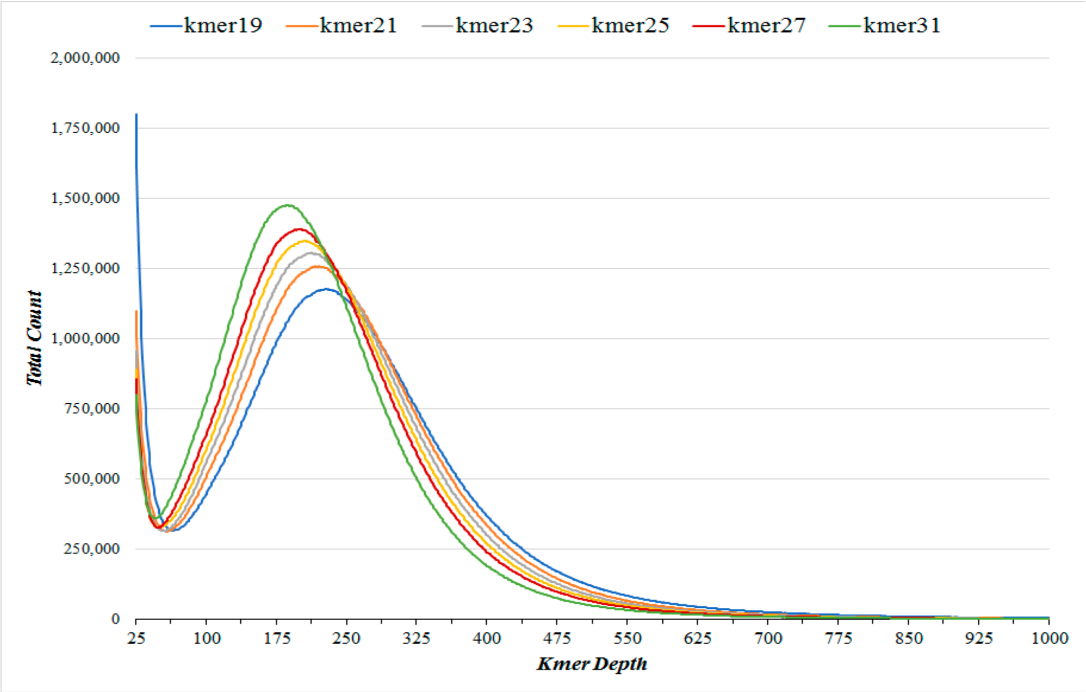

Plot of k-mer counts in the WGS Illumina pair-end sequences. After counting all 19-mers to 31-mers in the reads, the number of distinct k-mers (y-axis) that occur exactly X times (x-axis) were plotted. Histograms of k-mer frequencies in the raw read data for k = 19 (blue) and k = 31 (green). The extreme peak at k<25 on x-axis representing the distinct k-mers, is an artifact caused by sequencing errors. The peaks near x = 175 indicate the number of k-mers that occurred 175 times in the data, which correspond to regions where the assembler created distinct contigs for divergent putative haplotypes.

Figure S4: Dot plots display alignment of 10x Supernova contigs to the final hybrid scaffolds.

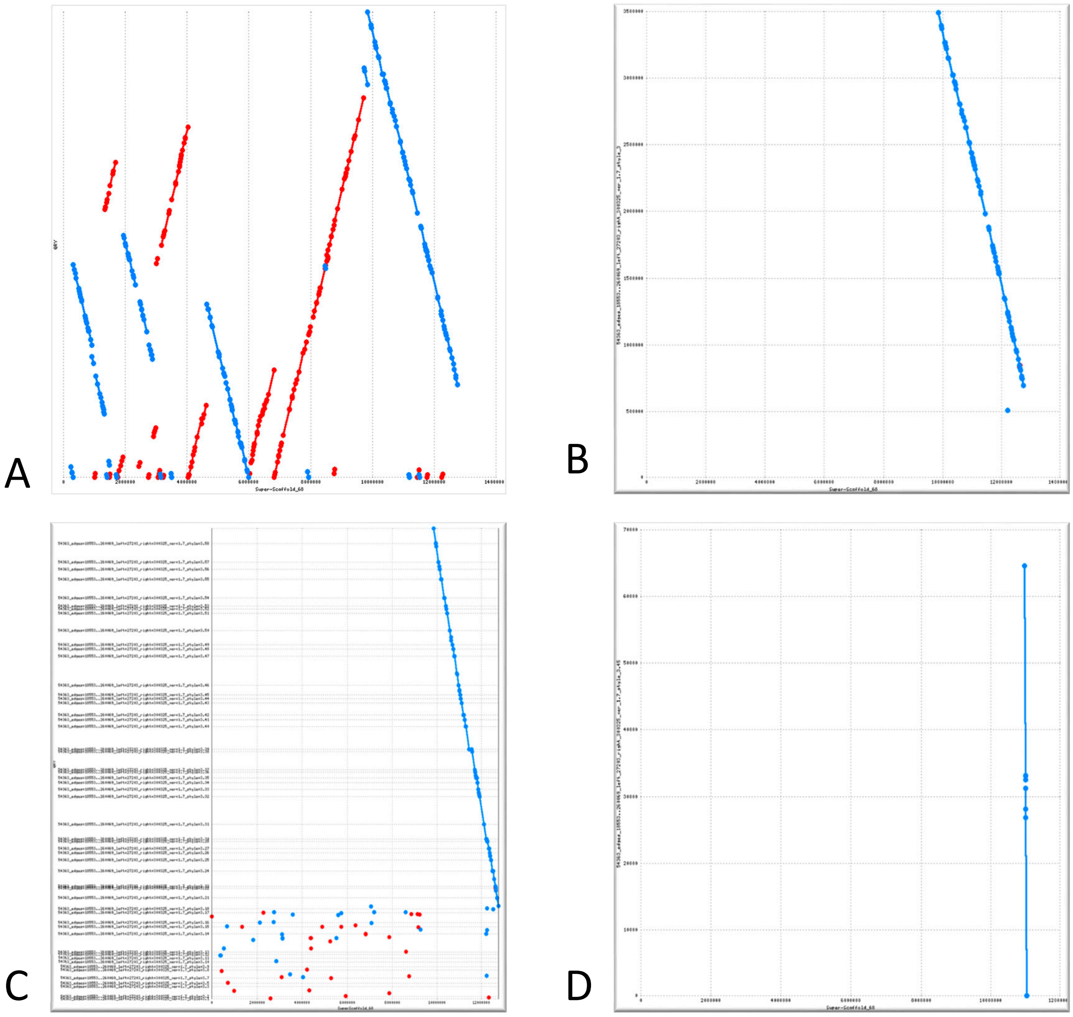

(A) shows alignment between biggest scaffold of the hybrid assembly, super scaffold 68 (13 Mb), and 10x Supernova contigs mapped to this scaffold. Blue lines and red lines denote the Supernova contigs mapped to forward or reverse strand of the super scaffold 68 sequence.

(B) shows alignment between hybrid assembly super scaffold 68 (X axis) and Supernova scaffold 54363 (3.4 Mb) (Y axis)

(C) shows alignment between super Scaffold68 and the contigs of Supernova scaffold 54363

(D) shows alignment between Super Scaffold68 and one of the contigs of Supernova scaffold 54363

Figure S5: Flow cytometric analysis of DNA content of insect cell lines.

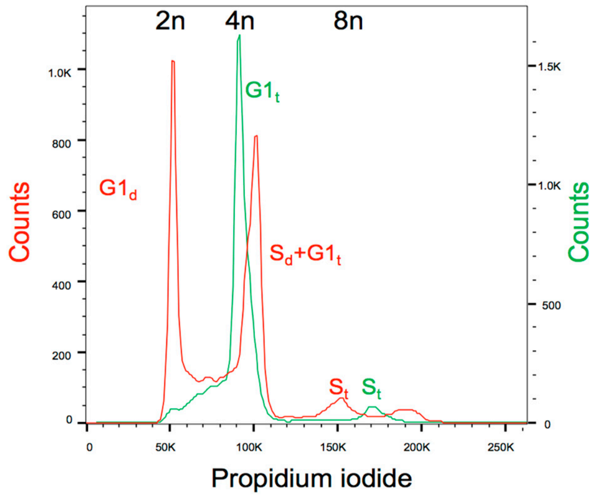

Shown are the *Spodoptera frugiperda* Sf9 cell line (red) and *Trichoplusia ni* Tni-FNL cell line (green). Based on fluorescence of propidium iodide, peaks are assigned to diploid (d) or tetraploid (t) DNA content for the various cell cycle phases.

Figure S6: Image and karyotype of Tni-FNL cell line.

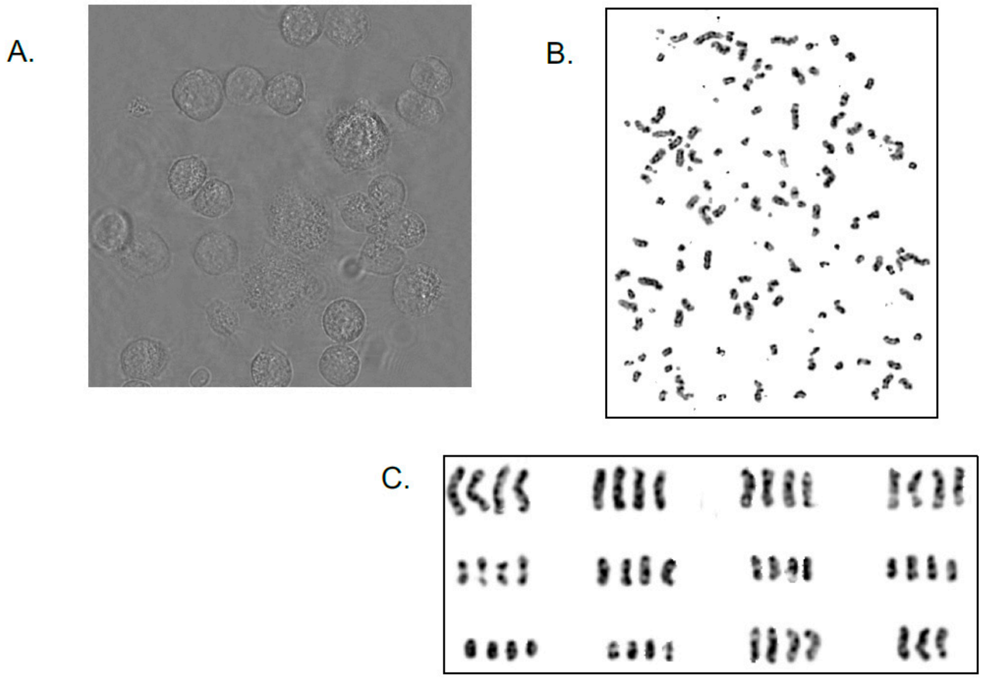

(A) Tni-FNL cell image taken on Nikon spinning disk on 35 mm plate with collagen.
(B) A chromosome spread from a representative Tni-FNL cell.
(C) A series of paired chromosomes from a single spread, based on banding patterns from GTG staining. In this case at least 11 sets of tetraploid chromosomes were able to be paired with reasonable accuracy

Figure S7: GC content and repeat elements comparison among three genome sequences.

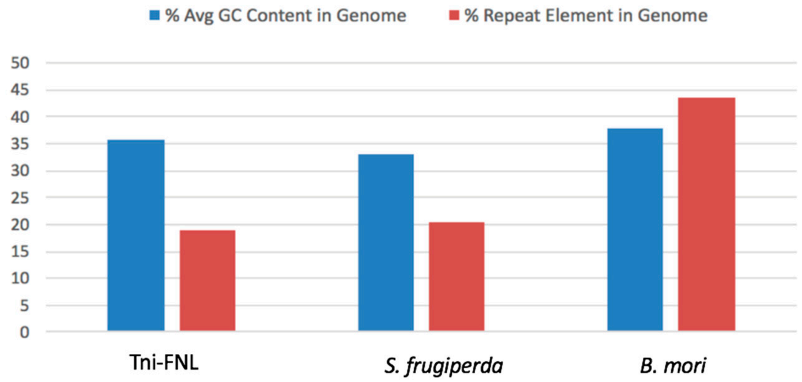

Comparison of the GC content and repeat elements among Tni-FNL, *S. frugiperda* and *B. mori* genomes

Figure S8: Identification of CpG islands in the Tni-FNL genome sequence.
(A)

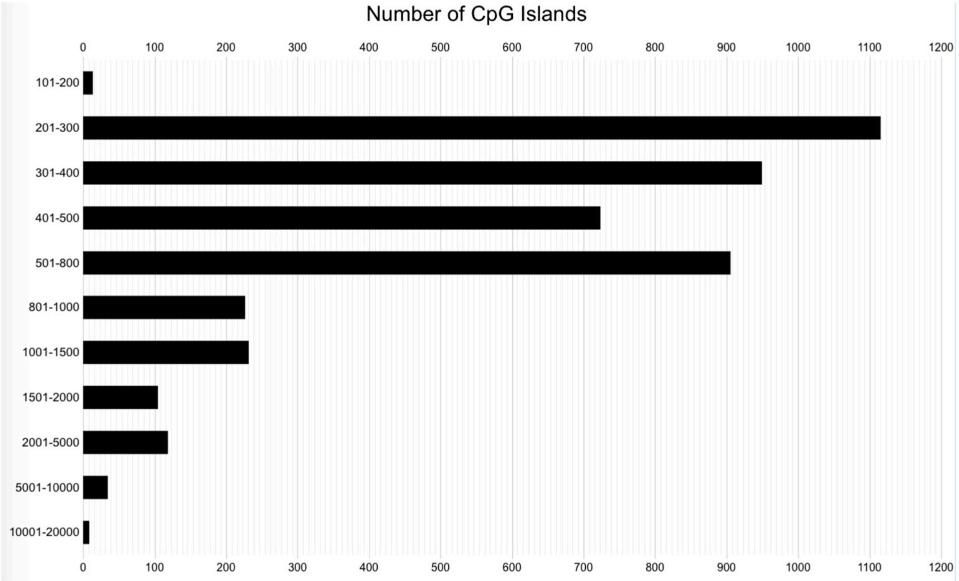

(A) Plot of length distribution of identified CpG islands in the Tni-FNL sequence in each size bin (y-axis) and CpG islands counts (x-axis).

(B) Distribution of CpG islands

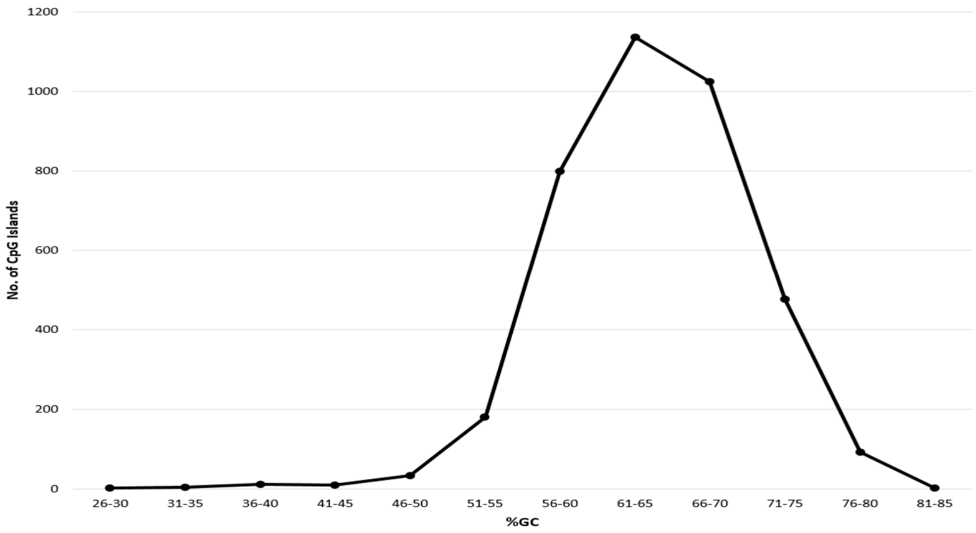

(B) Distribution of CpG islands vs. %GC content

Supplementary Tables

Table S1. Data generated from three different technologies.

Technology/Platform	Library	DNA Fragment Size (Mean)	Total Raw Yield (Gb)	Assembled Data (Based on Estimated Genome Coverage)
PacBio SMRT RSII	Pacbio 20 kb library, Swift Biosciences 20kb library	7.2 kb/Pacbio lib; 11 kb/Swift lib	30	110×
PacBio SMRT Sequel	Sequel 20 kb libraries, 2 SMRT cells (11Gb of 11 kb subread length)	11 kb	11	
10 Genomics Linked Reads /NextSeq 500	Chromium Genome library (60–100kb)	103 kb	121	338 × (total); 42 × (sub-sampled)
BioNano Irys Optical Maps	Irys Optical Map library Nicking enzyme used Nb.BssSI	100–2,000 kb	155	62×

Table S2. Repeat elements identified in the Tni-FNL genome sequence.

Elements	Subcategory	Number of elements*	Length occupied sequences (bps)	Percentage of sequence
SINEs		537	30,202	0.01%
	ALUs	0	0	0.00%
	MIRs	138	9,119	0.00%
LINEs		33,390	9,149,691	2.55%
	LINE1	53	3,167	0.00%
	LINE2	5,970	2,572,788	0.72%
	L3/CR1	1,238	345,607	0.10%
LTR elements		1,131	1,468,798	0.41%
DNA elements		2,986	1,746,214	0.48%
Unclassified		328,129	48,798,587	13.59%
Total interspersed repeats		366,173	61,193,492	17.04%
Small RNA		1,221	140,963	0.04%
Satellites		7	367	0.00%
Simple repeats		127,344	5,203,605	1.45%
Low complexity		22,648	1,041,779	0.29%
Total repeat region		517,393	67,580,206	18.82%

* most repeats fragmented by insertions or deletions have been counted as one element.

Table S3. Gene ontology classification of the genes predicted from the Tni-FNL genome assembly.

Species Name	Count Type	Biological Processes	Molecular Functions	Cellular Components
**Tni-FNL**	All count	17,656	40,834	8,025
	Species unique	884	952	316
Bombyx Mori	All count	25,148	61,316	11,730
	Species unique	930	991	316
Drosphila Melanogaster	All count	33,752	78,768	15,771
	Species unique	939	994	322

Table S4. Comparison of shared GO category genes from Tni-FNL, *B. mori* and *D. melanogaster*.

Compared Pair of Species	Biological Processes	Molecular Functions	Cellular Components
Tni-FNL & *Bombyx Mori*	858	928	305
Tni-FNL & *D. Melanogaster*	818	907	298
D. Melanogaster & *Bombyx Mori*	859	931	298

Table S5. Transcript structure comparisons between Tni-FNL, *S. Frugiperda* and *B. mori*.

	**Tni-FNL**	***S. frugiperda***	***B. mori***
	**Exon**		
**Total number of exons**	105,550	64,725	197,632
**# exons/transcript (Mean)**	7	5.58	8
**Longest Exon**	13,780	12,798	11,884
**Mean Exon Length**	298	245	298
**Shortest Exon**	3	3	1
**Longest Intron**	452,085	15,320	528,292
**Mean Intron Length (bp)**	989	726	2,931
